# Meetings of Societies

**Published:** 1925-01

**Authors:** 


### MEETINGS OF SOCIETIES.

### Bristol Medico-Chirurgical Society.

The Annual Meeting of the Society was held in the Physiological Lecture Theatre of the University on October 8th, 1924, the President and seventy-three members and visitors being present. Drs. G. A. G. Gee and T. C. Brentnall were elected members.

The President, Mr. J. L. Firth, in resigning the chair to the in-coming President, Dr. J. O. Symes, alluded to the loss the Society had sustained during the past year by the death of Dr. L. M. Griffiths, late Honorary Medical Librarian and Assistant Editor of the *Journal*, Mr. R. G. P. Lansdown, late President, Mr. Forbes Fraser, C.B.E., and Mr. Farr, librarian of the Society's library. He mentioned that a fund had been started for the assistance of the widow and children of the latter, which he commended to the notice of members.

A vote of thanks was passed to Mr. J. L. Firth for his services to the Society during his year of office.

The in-coming President then took the chair and delivered his inaugural address. (See page 1.)

The Honorary Secretary presented his report and balance sheet, and read a letter from the Vice-Chancellor of the University accepting the Library of the Society, and expressing the thanks of the Council of the University for this gift. Mr. S. V. Stock was then re-elected Honorary Secretary for the ensuing year.

The Editorial Secretary, Dr. A. L. Flemming, read his annual report and balance sheet. A vote of thanks was passed, and he was re-elected.

Mr. T. C. Carwardine was chosen President-elect.

The following members were elected members of the Committee :—

*Past Presidents.*—Mr. F. Lace, Dr. J. A. Nixon, C.M.G.

*Members.*—Dr. D. C. Rayner, Mr. C. F. Walters, Professor E. W. H. Groves, Mr. A. J. M. Wright, Dr. H. L. Ormerod, Dr. W. V. Wood, M.C.

Dr. C. K. Rudge was re-elected Honorary Medical Librarian.

Drs. G. Parker and W. A. Smith were elected representatives of the Society on the University Library Committee.

---

*November 12th,* 1924.

J. Odery Symes, M.D., President, in the Chair.

Mr. Wilfrid Adams showed cases illustrating "**Surgical Nerve Lesions in Children.**" (See p. 32.)

Mr. Richard Warren read a paper on "**Appendicitis Causing Intestinal Obstruction.**" (See p. 23.)

---

*December 10th,* 1924.

J. Odery Symes, M.D., President, in the Chair.

Dr. C. E. K. Herapath and Mr. C. F. Walters showed a case of "**Acholuric Jaundice.**"

The patient, a male aged 38, had suffered from jaundice for eleven years, with progressive pallor ; during four attacks of more severe jaundice there was pain and vomiting. The liver was moderately enlarged, the spleen very much enlarged. Examination of the blood showed R.B.C. 2,400,000, hæmoglobin 27 per cent., W.B.C. 6,400, polychromatophilia, anisocytosis, Wassermann negative, serum contained 15 units of bilirubin. The urine contained much urobilin, no bilirubin. The stools contained bile. Tests (lævulose) showed a deficient liver function. A diagnosis was made of hæmolytic jaundice, and Widal's differential test indicated the acquired type. The spleen was removed by Mr. C. F. Walters. It weighed 1,200 gm. Nine days after the operation the blood showed R.B.C. 3,960,000, hæmoglobin 70 per cent., W.B.C. 29,000, normal cell appearance. Dr. HERAPATH referred to the present conception of the pathogenesis. Two factors were concerned : (1) increased fragility of the corpuscles ; (2) presence of abnormal agglutinins. (A full account of the case will appear in the *British Medical Journal.*) Mr. C. F. WALTERS described the steps of the operation. Slight cases did not require surgical treatment ; but in all severe cases this was necessary. The operation should be carried out during the intervals between crises ; the dangers were small, and the results good. The PRESIDENT asked whether this was true acholuric jaundice. The fragility of the corpuscles did not seem typically great. He remarked that four points needed care at operation : (1) Separate operation to remove gallstones, (2) the spleen must be movable, (3) the operation must be rapidly completed, (4) vessels must be tied close to the spleen. Mr. A. R. SHORT said he had seen one case of each type : (*a*) Congenital in a child, which did not need operation ; (*b*) acquired, in a woman of 40. He removed numerous bilirubin gallstones ; but trouble continuing, he removed the spleen ; the patient died. He referred to experimental work at the Mayo clinic, in which it had been shown that after removal of the liver jaundice occurred (from the action of the spleen). Dr. V. M. COATES, M.C., said he had seen several cases of acquired jaundice of this type. He asked what experience Dr. Herapath had had of liver efficiency tests (*e.g.* Widal's, glycuronic, etc.) other than that described (Van den Berg's lævulose test). Dr. KENNEDY had seen one case occurring after concussion. Was there a connection between this and pernicious anæmia which sometimes followed head injuries ? Possibly the hæmolytic cells in the liver and spleen were under nervous control. Dr. HERAPATH, in reply, said that he considered the two types to be one disease. In the case shown the fragility of the corpuscles was definite though not marked, and not altered by operation. The lævulose test was the only one he had used. He did not think the brain influenced the activity of the reticular cells of the spleen.

DR. C. E. K. HERAPATH showed a case of "**Bronchiectasis in a child of six.**" This girl had had an abdominal operation, followed by pneumonia. Signs pointed to a cavity in the left base ; but when the question of surgical treatment was raised a cavity was also found in the right lung. Mr. C. F. WALTERS said that he did not propose to operate in this case. He reviewed the developments in the surgery of the condition. (1) Incision, offered no prospect; (2) thoracoplasty, offered no prospect; (3) treatment via the bronchoscope was an improvement; (4) removal of a lobe of the lung had a mortality of 50 per cent.; (5) the latter procedure as a two-stage operation had been tried once with success; (6) the best method so far seemed the cautery after "exteriorisation," eleven cases reported, with one death. Mr. A. J. Wright considered that bronchoscopy might be useful. It was not a very difficult or dangerous method. Dr. D. A. Alexander advised the use of creosote baths, of which he had had favourable experience.

Mr. A. J. Wright read a paper on "**Carcinoma of the Œsophagus (including the deep pharynx).**"

Mr. Wright reviewed the incidence of œsophageal cancer. It was usually seen in males after middle life, and affected the upper and lower parts of the tube equally. In women, and almost exclusively in women, postcricoid carcinoma was found, and occurred on the average somewhat earlier than the types affecting the other sex.

*Ætiology.*—He directed attention to the causes thought to influence carcinoma of the tongue, an analogous part; very little was actually known of the predisposing factors in the œsophagus. (1) Syphilis appeared to be very rare in the latter region. (2) Trauma was occasionally associated with a subsequent growth; especially the trauma of hot liquids had been suggested as a reason for the incidence of postcricoid growths in women. (3) Sepsis may be a factor, as for example that associated with pyorrhœa.

*Symptoms.*—The only symptom of importance was difficulty in swallowing. This may appear with startling suddenness. It is *not* a sign of aneurysm, which in the speaker's experience never gave rise to real difficulty in swallowing. Cough was common in postcricoid growths. Coughing up of food occurred when an œsophageal-tracheo fistula had formed; this condition seemed compatible with life for at least some months.

*Diagnosis.*—Laryngoscopy might assist, especially in cases of postcricoid growth. Skiagraphy was of great value; the findings were difficult to interpret when the obstruction was high. Œsophagoscopy would usually clinch the question.

*Treatment.*—Operation held little hope of success. Of 70 cases collected he had found 65 resulted in death. Radium applications appeared to afford some hope, and usually improved the condition for a time, though the dilatation might be in part responsible for this. The position was unfavourable for deep X-rays, of which he had no experience. As palliatives the introduction of a tube seemed the best. Dilatation alone helped only for a short time. Various types of tube were employed; he thought Souttar's tube would be found the best. He had in three cases used diathermy, with gratifying results. Finally he must mention gastrostomy; this was not a satisfactory operation in the hospital type of patient.

Mr. Wright demonstrated specimens of different types of tube for intubation, and also his own electrode for diathermic applications.

The PRESIDENT had observed the frequent occurrence of cases of carcinoma of the œsophagus with a very long history of dysphagia. As to treatment, he had had a good result in one case after using radium, relief lasting twelve months. Mr. J. P. I. HARTY had also seen several cases with a long history of some difficulty in swallowing. And these were not all retrocricoid growths. He agreed that X-rays did not much assist the diagnosis in retrocricoid growths. Of the latter, his experience of operation was that it was not a good method of treatment. He asked what amperage Mr. Wright used for diathermy, and for how long the current was applied. He had had one case in which he had used Souttar's tube; the result was not good, the tube being returned. He advised intubation especially for strictures which were firm, not for the fungating type. Dr. C. E. K. HERAPATH was interested in the suggestion that drinking tea might be a factor in producing postcricoid cancer. What was the effect of drinking whisky? He had never seen a case of aneurysm produce dysphagia. Mr. C. F. WALTERS thought the main benefit of radium was due to the dilatation. As for gastrostomy, he found that at least in private practice this measure was often very helpful. Mr. A. R. SHORT had had three cases for which he had used Souttar's tubes with encouraging results. He was very reluctant to advise a gastrostomy. Mr. LACY FIRTH thought that Trotter's operation was a good one for postcricoid growths. He considered that for the majority of cases gastrostomy was the best palliative. Mr. E. WATSON-WILLIAMS said apropos of "cures" following the use of radium, that Dr. Guisez's experience was that the commonest type of growth was a basal cell carcinoma; he wondered whether this was the experience also of others. Dr. C. CORFIELD mentioned, in reference to ætiology, that in China, where the men eat before the women and get their food hotter, cancer of the œsophagus was more common in men. In reply, Mr. WRIGHT said that there were cases in which a long history of dysphagia was probably due to a congenital narrowing of the pharynx. He had not considered operations on the postcricoid region as included in the subject. He had no rule as to the amperage he employed in the œsophagus nor as to length of application.

---

*January 14th, 1925.*

J. Odery Symes, M.D., President, in the Chair.

Mr. C. F. Walters showed four cases of surgical interest:

1. Compound fracture and dislocation of the astragalus; conservative treatment had saved a useful foot.

2. Diverticulitis of the colon, with skiagrams.

3. Actinomycosis of the abdominal wall, appearing one year after removal of appendix; treated with iodine internally.

4. Specimens of primary carcinoma of the small intestine.

In opening a discussion on "Encephalitis Lethargica," Dr. Edgeworth said that, from figures supplied to him from Dr. D. S. Davies, the M.O.H., it appeared, that in the five years 1919–1923 151 cases of lethargic encephalitis had been notified in the City and County of Bristol. Of these 13 could not be traced. Of the remainder, 138 cases, 68 had died and 70 had recovered more or less completely, a mortality of 49 per cent. Of the 70 cases recovering 26 had recovered completely, whilst 44 presented residual phenomena. The figures for 1924 showed great changes; 158 cases had been reported, of which 27 had died. The disease had become more widespread, but with a far less mortality—17 per cent. The causes of this difference in the mortality might be due, in fact, to a better acquaintance by doctors with the clinical features of the disease, but possibly it was due to a diminished virulence of the infecting organism. It had yet to be determined whether this diminished mortality is associated with a diminished incidence of residual phenomena. Dr. Edgeworth then gave a sketch of the chief phenomena of the disease. He pointed out that whereas it was known that the Parkinsonian syndrome might arise after an interval of health, (citing a case where the interval was two and a half years), it was not known whether other residual phenomena might similarly arise. Dr. D. S. Davies said that Bristol showed no difference from the rest of England as regards incidence and mortality. The disease was most frequent in April, and tended to occur in densely-populated areas. Dr. C. F. Coombs mentioned a prodromal period of "neuritic" pains seen in some cases, and suggested that it might eventually be possible to administer abortive treatment. He mentioned one accident on lumbar puncture, as a warning not to remove fluid too rapidly. Dr. H. H. Carleton described the diplopia as one for distant objects only. The lethargy might recur for periods of months after apparent recovery. He also had noted the period of pains just mentioned. For treatment he had formed a favourable opinion of mercury, but did not think hyoscine of value. Dr. R. A. Askins (Deputy M.O.H.) spoke of the absence of evidence of contagion. Late mental impairment among the cases that survived seemed common. Mr. E. R. Chambers confirmed Dr. Carleton's observation as to the character of the diplopia. No one muscle was concerned; he thought it due to a paralysis of divergence. Dr. John Wallace (M.O.H., Weston-super-Mare) pointed out that figures of incidence for such a community, where many people were sent to recuperate from illness, might be misleading; some developed the disease while visiting the town. Sudden vomiting might be the first symptom. Colonel A. E. J. Lister pointed out that cases in which optic neuritis occurred were not very rare. Dr. R. G. Gordon said that a common type in children showed lethargy only from early morning till noon; from then till evening the patients were normal, and through the night very restless. He mentioned that cases had occurred among miners where long after apparent recovery there was a persistent lack of initiative. In children a frequent feature of the residual phenomena was loss of moral control. Myoclonus, like the Parkinsonian syndrome, might recur after a long period of apparent recovery. He found hyoscine useful for sufferers from myoclonus, if given in large doses. The President (Dr. J. O. Symes) analysed the late results in 33 cases. 11 were dead; 2 were well; 10 at work, but suffering from tremors, spasms, etc.—such might come on even after three years from "recovery"; and 10 were showing definite psychoses of varying degree.

---

